# The role of bowel for minimally invasive treatment of stricture disease

**DOI:** 10.3389/fruro.2023.1080856

**Published:** 2023-03-14

**Authors:** Shane Kronstedt, Alain Kaldany, Hiren V. Patel, Sammy E. Elsamra

**Affiliations:** Division of Urology, Rutgers Robert Wood Johnson Medical School, New Brunswick, NJ, United States

**Keywords:** appendix, bowel, genitourinary reconstruction, stricture, robotic surgery

## Abstract

The management of urinary tract stricture disease has evolved over the last several decades, with robotic surgery representing a bourgeoning method for urologic reconstruction. Conventionally, proximal and mid-ureteral strictures, as well as lengthy urethral strictures, have presented a challenge for surgeons to create tension-free repairs. Options for repair include endoscopic dilation, endopyelotomy, ureteroplasty or pyeloplasty, and urethroplasty. Small and large bowel can be incorporated into various urinary tract stricture repairs. Their use has proven successful in reconstructing both upper and lower urinary tract strictures and offers flexibility for complex cases such as lengthy or multifocal strictures. While the use of bowel, most notably the appendix, for stricture repair is not a novel concept, a growing body of evidence supports its use with minimally invasive robotic approaches. In addition, there has been a substantial amount of recent data suggesting low rates of postoperative complications and long progression-free survival after robotic stricture repair using small bowel or rectum. We present a comprehensive review of literature outlining the role of the small bowel, appendix, and rectum in the minimally invasive repair of urinary tract stricture disease, as well as a description of the various techniques employed.

## Introduction

A functional obstruction characterizes Genitourinary (GU) stricture disease due to narrowing of the ureter in the upper urinary tract or urethra in the lower urinary tract. The management of GU stricture disease has seen an evolution over the last several decades that advances in the robotic surgical platform have further propelled.

The use of bowel has remained an essential staple in the armamentarium of reconstructive urologists. Bowel represents an abundant and readily available autologous tissue source with a rich bloody supply and mucosal layer adequate for anastomosis. For upper tract stricture repair, using a bowel graft instead of a buccal mucosa graft, for instance, has the added benefit of a single surgical site, reducing the morbidity associated with secondary harvest sites. Recently, unique applications of bowel use have shown clinical success in managing upper and lower urinary tract stricture disease. This review outlines the use of bowel as a versatile tool for managing GU stricture disease and highlights innovative applications that have been recently utilized.

## Ureteral stricture disease

Ureteral strictures are characterized by narrowing of the ureter, which leads to a functional obstruction (1). As urine drainage becomes restricted, urinary stasis results in the upper tract and renal pelvis, leading to flank pain, upper tract infections, and potentially renal failure (2–4). There are various etiologies of ureteral strictures, including radiation, iatrogenic injury, trauma, urolithiasis, and congenital causes (5). They can be classified as ureteropelvic junction obstruction (UPJO) or proximal, middle, distal, and pan-ureteral strictures. Longer or more complex ureteral strictures require more advanced surgical techniques, such as renal mobilization with downward nephropexy, ileal ureter replacement, transureteroureterostomy, and autotransplantation of the kidney to provide a tension-free anastomosis (1).

Over the last several decades, the technical aspect of management for ureteral stricture disease has evolved, with robotic surgery becoming the preferred method. Robotic-assisted laparoscopic techniques have recently led to significant advances in treating ureteral strictures and have significant benefits compared to laparoscopic or open surgery. Nezhat and colleagues first described laparoscopic surgery for urologic reconstruction in 1992 by performing a laparoscopic ureteroureterostomy (UU) (6). Still, this procedure was not widely adopted due to the technical challenges of laparoscopic suturing in a limited operative field. Robotic surgery for treating ureteral strictures was first described in 2003 when Yohannes and colleagues performed a robotic-assisted laparoscopic ureteral reimplantation with a Boari flap to treat a distal ureteral stricture (7). Robotic surgery has the advantages of three-dimensional vision, improved visualization, and higher degrees of mobilization due to robotic wrists. This has led surgeons to utilize it more frequently over the past decade for the surgery of mid and proximal ureteral strictures, with some considering it the standard of care (5). The robotic-assisted laparoscopic (RAL) approach has shown high success rates in ureteral repair, similar to open surgery, with the added advantage of faster recovery after surgery (8).

## Upper urinary tract stricture repair

Stricture disease in the upper urinary tract poses a unique challenge for urologists, given the anatomical considerations of the ureter and the surgical field. Urologists must be familiar with various techniques depending on the ureteral stricture’s etiology, length, and location. Strictures in the proximal ureter can be managed by ureteroureterostomy, transureteroureterostomy, graft interposition or onlay, or ureterocalicostomy. Longer strictures or those in the mid-ureter may warrant reimplantation with Boari flap, auto-transplantation, or bowel substitution. Strictures in the pelvic ureter are often treated using reimplantation alone, with the option to include Boari flap or psoas hitch for added length (3).

Ileal substitution is a long-established technique, though it has been associated with multiple complications, including urinary tract infection, mucus in the urine, increased absorption of urine, and electrolyte imbalances (9). Using the appendix has a lower risk of metabolic consequences than the ileum, as there is minimal reabsorption of urinary solutes due to its lower surface area and because the appendix does not play a significant role in electrolyte transport (10). Additional benefits include a similar diameter to the native ureter and lack of bowel anastomosis needed for harvest (11). Evidence suggests that the appendix does not provide peristaltic propulsion; directionality and positioning will not promote or hinder urine flow (12–15). Prohibiting factors for the use of appendix include inadequate length, prior appendectomy, and appendiceal fibrosis from inflammation or radiation (11).

The minimally invasive harvest typically uses a laparoscopic stapler to excise the appendix. Once the appendix is harvested, its two ends are opened, and the lumen is copiously irrigated in preparation for implantation. When using the appendiceal onlay technique, the ureteral blood supply is minimally disrupted, which supports anastomotic healing and the success of the repair. In the appendiceal interposition technique, the blood supply is divided between the proximal and distal portions of the ureter. Both methods are effective. Still, the appendiceal onlay is considered a superior technique because its blood supply remains intact when transferred to the tissue (5). In a study of 4,680 post-mortem specimens, the appendix’s average length was 8.21 cm (16). This length provides a significant amount of tissue graft but is limited in bridging more significant ureteral defects and left-sided strictures; the appendix is exceptionally advantageous for right-sided ureteral repair (9).

### Appendiceal interposition

The ureteral appendiceal interposition (UAI) is an advantageous technique for ureteral strictures, particularly right-sided or completely obliterative strictures. After the first reported appendiceal interposition in 1912, most cases using UAI have either been single cases or reports of small series within the pediatric population (17). Few cases have been reported using the appendix for complex ureteral stricture repair in the adult population. Most have been performed with the appendiceal onlay technique rather than ureteral substitution. The UAI approach is considered a viable option for treating complex ureteral stricture disease when using alternatives to buccal or ileal mucosa and is particularly beneficial in cases where the onlay technique is not feasible (e.g., ureteral obliteration) (11). In a retrospective review of 11 patients by Burn et al., UAI was shown to be safe and effective in treating ureteral stricture disease (11).

In this technique, a robotic or open approach can replace the diseased segment of the ureter with the appendix. The ureter is transected, the stenotic section excised, and the appendix is sewn between the proximal and distal transections of the ureter. The appendix is not detubularized and, as such, utilizes the appendiceal lumen for the passage of urine.

Multiple examples of success with the appendiceal interposition technique have been described in treating traumatic ureteral injury, radiation-induced stricture disease, obliterative stricture disease, and pediatric stricture disease (18–23). In 2000, Richter and colleagues performed an appendiceal interposition to replace the ureter in three children. They showed successful outcomes with no evidence of obstruction and stable renal function (22). In 2005, Mhiri et al. described six cases of long ureteral loss treated with an appendiceal ureteral interposition (24). They showed a recovery of kidney function in all cases. In 2020, Burns and colleagues looked at surgical outcomes of 11 patients with obliterative ureteral strictures measuring > 2 cm who underwent UAI (11). No patients required any repeat interventions due to the recurrence of their stricture disease. However, three patients (27%) had complications that required procedural or surgical interventions, two of which had prior radiation. The rates showed similar initial success rates compared to buccal mucosal onlay repair, with success rates of ~83% and complication rates of ~16% (11).

### Appendiceal onlay

**Figure 1:**
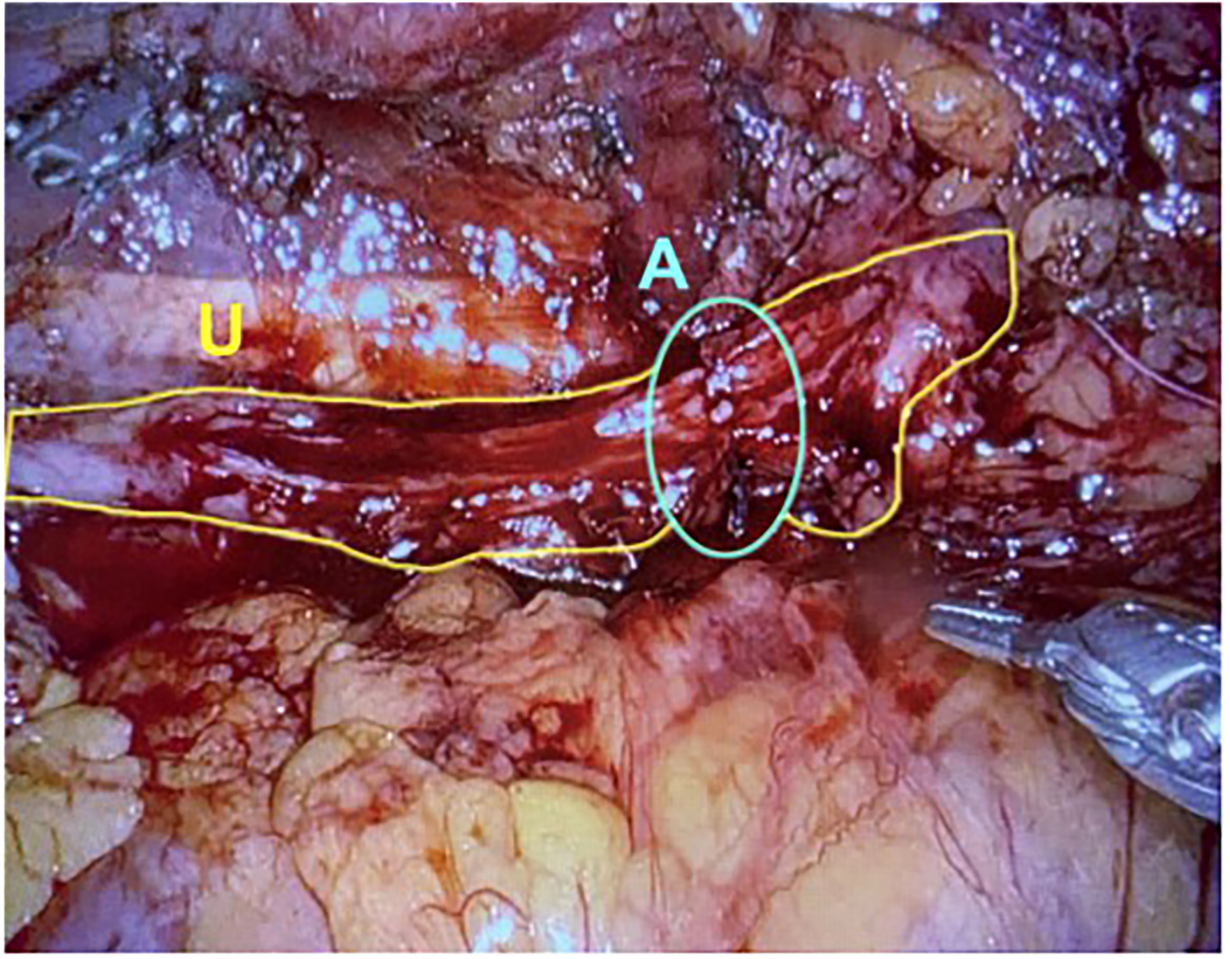
The ureter is excised longitudinally, leaving a plate of ureter available for the onlay of graft tissue. U, Ureter; A, Anastomosis. (Images courtesy of SEE.).

**Figure 2:**
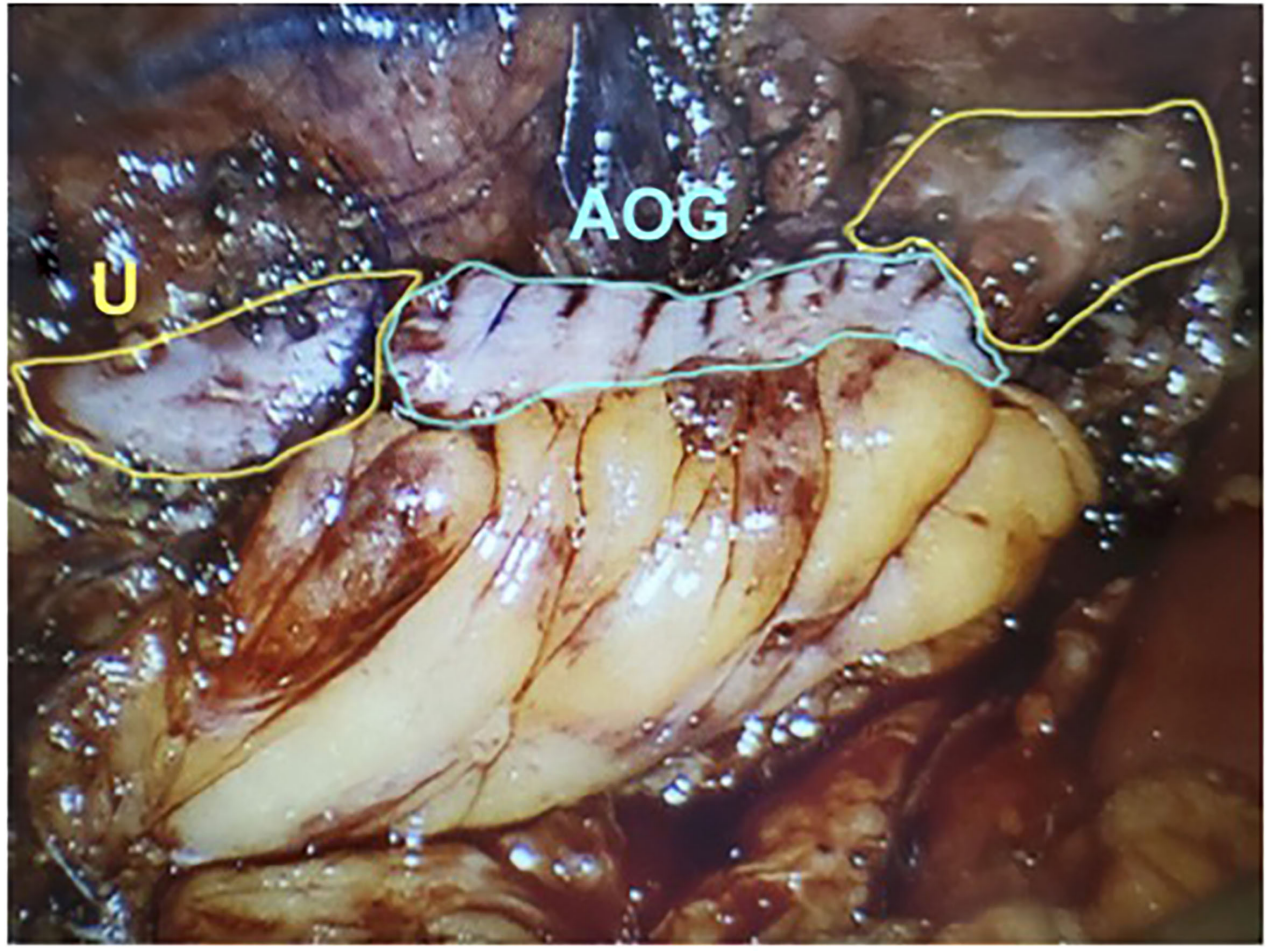
The detubularized appendix is sewn to the ureteral plate and anastomosed to both aspects of uereter. U, Ureter; AOG, Appendiceal Onlay Graft. (Images courtesy of SEE.).

In 2009, Reggio et al. reported the success of a superior technique in treating a nonobliterative right ureteral stricture by performing the laparoscopic appendiceal onlay flap ureteroplasty (9). In this technique, the ureter is not completely divided but rather incised longitudinally, leaving a “ureteral plate” in place with minimal destruction of the blood supply (**Figure 1**) (11, 25). The appendix is sewn to the ureterotomy to augment the luminal diameter (**Figure 2**) (11). The appendiceal flap ureteroplasty provides many advantages, including the relative ease of appendiceal mobilization, a well-defined blood supply, negligible absorption of urine over a small surface area, allowing for a tension-free anastomosis, and a lack of donor site morbidity compared to a buccal mucosa graft (BMG) ureteroplasty (5). The appendiceal onlay technique is beneficial for treating radiation-induced strictures or any other pathology with compromised vascularity since the appendiceal flap carries its blood supply and provides minimal disruption to the ureteral vessels (5).

In 2015, Duty et al. showed no recurrence in a case series of six patients at 16 months of follow-up (26). All the strictures were right-sided and had an average length of 2.5 cm (5). This technique has also been employed to repair an obliterative right ureteral stricture measuring 5 cm, with no recurrence at ten months (11). More complex repairs are also possible. For instance, an 11cm panureteral appendiceal ureteroplasty for an iatrogenic 15 cm right ureteral avulsion was recently performed, which required a downward nephropexy, psoas hitch, and calycostomy. At six months, renal function was preserved, and no obstruction was seen (27).

### Ileal ureter substitution

The first ileal ureteral substitution was described in 1906 by Shoemaker and later popularized in 1959 by Goodwin et al. (28, 29) In the 1990s, Yang and Monti refined the technique to treat longer strictures (30, 31). The original ileal ureters were used to treat tubercular obstruction. However, broader indications have been expanded into use recently, with both the RAL and intracorporeal approaches used as acceptable techniques (5). In a review of 17 ileal ureter series and 387 patients, the most common indication was a stricture following a urologic procedure (22.0% of cases) (32).

Ileal ureter substitution is primarily used as a fallback technique when other less-aggressive techniques are not feasible (5). Typically, it is used for ureteral replacement in cases of long segmental defects and complex pathophysiology that is not amenable to other simpler techniques (11). The use of ileal ureter substitution comes at the cost of multiple complications; metabolic abnormalities and elevated serum creatinine can be seen due to its resorptive nature. Other potential complications include bowel obstruction, fistulae, bowel leak, and long-term metabolic consequences such as metabolic acidosis, B_12_ malabsorption, and bile acid malabsorption leading to nephrolithiasis or cholelithiasis (5). This technique is usually contraindicated in patients with inflammatory bowel disease, bladder outlet obstruction, neurogenic bladder, and short gut syndrome (33).

**Figure 3:**
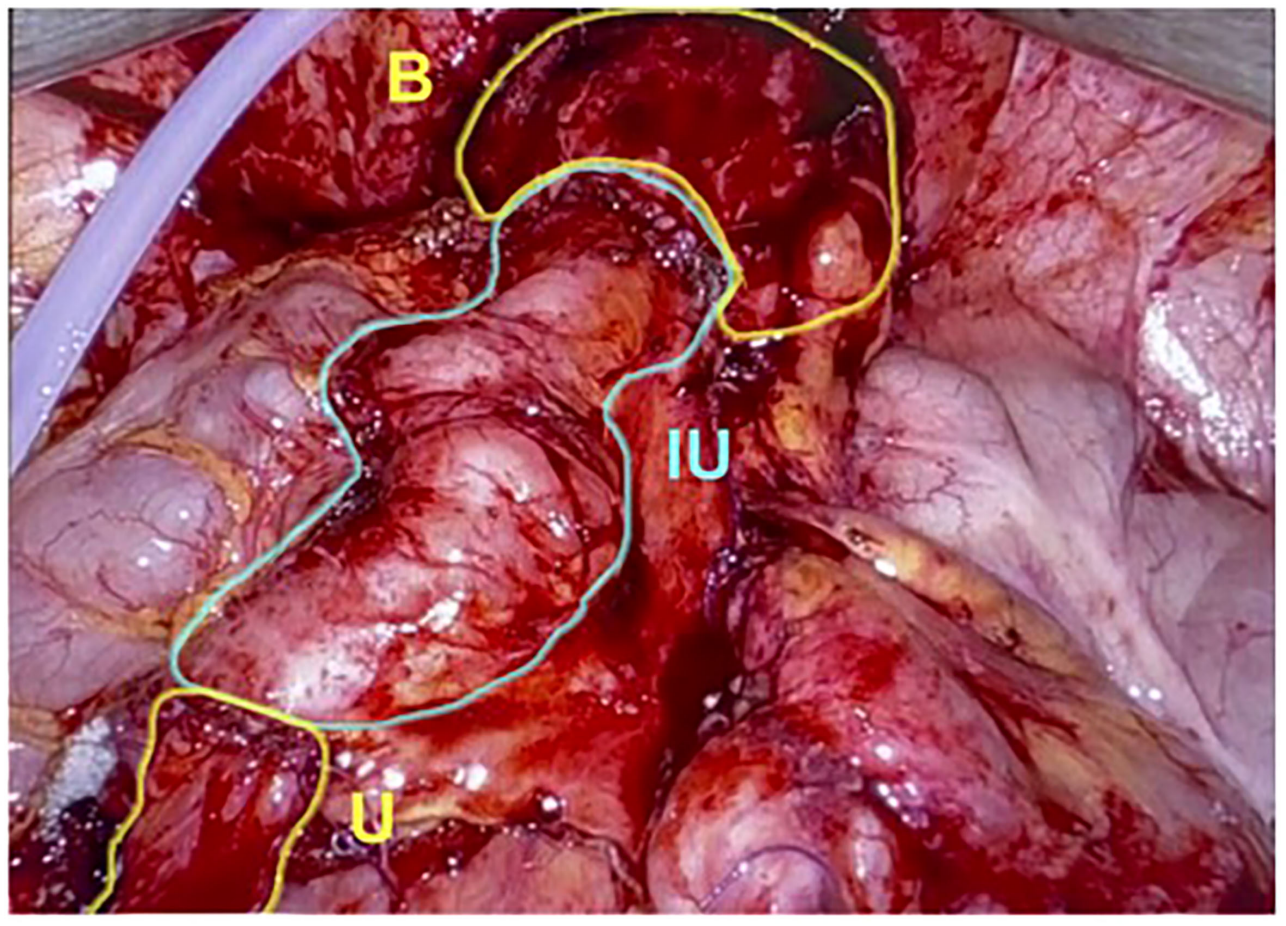
The graft of ileum is used as an interposition to create a connection directly between the native ureter and bladder. U, Ureter; IU, Ileal Ureter; B, Bladder. (Images courtesy of SEE.).

In this technique, the ureter is isolated, and the patent end of the ureter, bladder, or renal pelvis is exposed. Approximately 20 cm of ileum proximal to the ileocecal valve is harvested with a laparoscopic stapler, and bowel to bowel anastomotic continuity is reestablished appropriately. Proximally, the bowel can be anastomosed to the ureter, renal pelvis, or lower pole calyx, depending on the severity and location of the stricture. Distally, the end of the ureter can be anastomosed to the bladder or a spatulated distal ureteral stump (**Figure 3**). Bilateral ureteral strictures can be treated by harvesting a longer segment of the bowel and performing ureteral anastomoses on both ends, forming a U configuration with the most dependent portion of the bowel segment overlying the bladder. An anastomosis is then made between an enterotomy on the antimesenteric side of the ileum and a matching cystotomy on the dome of the bladder (5, 34).

Other advantageous techniques have been described, such as the Yang-Monti tube, which involves reconfiguring a short and wide intestinal segment (typically distal ileum) into a long, narrow tube used for ureteral repair. With this technique, a 2-3 cm segment of ileum can provide 8-10 cm of reconfigured length. One advantage of the Yang-Monti tube is the ability to create a nonrefluxing anastomosis. It is typically unnecessary, but if reflux prevention is desired, a nonrefluxing anastomotic technique can be employed, such as the Leadbetter-Politano or Lich-Gregoir techniques (34).

In 2009, Armatys and colleagues (32) conducted a single-institution retrospective analysis of 91 patients who underwent ileal ureter replacement. The mean length of the ileum was 14.2 cm (range 4-35). Short-term complications were seen in 42.9% of patients. However, serum creatinine decreased or remained stable in 74.7% of patients. In 2016, Chopra and colleagues described a three-case series where 2 out of 3 patients had a successful outcome (35). In 2018, Ubrig and colleagues reported a seven-patient series using a robotic intracorporeal approach. Five patients received a simultaneous psoas hitch, and the mean length of ileum was 20.4 cm. All patients were transplanted successfully and symptom-free three months after surgery (36).

## Lower urinary tract/urethral stricture repair

Short-segment urethral strictures can be reconstructed with endourologic techniques or open procedures, including dilation, urethrotomy, and anastomotic urethroplasty, which can be further augmented with suspensory ligament mobilization, crural separation, inferior pubectomy, and corporal transposition (37). Unlike proximal strictures, distal urethral strictures are typically managed with reimplantation, achieving additional length from an augmentation urethroplasty, such as a dartos flap, penile skin flap, or buccal mucosal graft (37). More recently, the use of bowel has been increasingly described to treat complex stricture disease for both the upper and lower urinary tract.

Repairs of lower urinary tract strictures have traditionally used non-hair bearing skin, such as buccal mucosal grafts (BMG), to make tunical flaps for urethral reconstruction. The advantages of BMG include its thick epithelium, accessibility, and robust data supporting long-term success rates. Still, there are cases where BMG may not be feasible given increased length requirements warranting more tissue, such as a urethral stricture after gender-affirming phalloplasty (38). In search of a suitable graft tissue for the urinary tract that is easily perfused, physicians have tried multiple graft sources, including penile, colonic, oral, bladder, and rectal tissue (39). Considering bowel, various minimally invasive approaches have been described to obtain sigmoid or rectal mucosa grafts (RMG), with promising outcomes regarding safety and efficacy (38, 40–42).

### Rectal mucosal graft

Rectal mucosal grafts (RMG) are ideal for patients who are not suitable candidates for a BMG harvest. Alternate grafts to BMG must be considered when larger quantities of tissue are required to graft long-segment strictures or when treating a recurrent stricture made from buccal mucosa. This is especially important for patients who have lichen sclerosis (40). Harvested RMG can measure up to 8 cm in diameter and are an excellent option for patients with long urethral strictures (43).

A multi-institutional analysis was conducted using the Trauma and Urologic Reconstructive Network of Surgeons (TURNS) database to look at outcomes of urethroplasties using RMG in thirteen patients (44). Most patients (69%**)** had failed a urethroplasty using a BMG. The median stricture length was 13 cm, and the mean RMG length used for repair was 10.6 cm (3-16). Various techniques were used to include dorsal and ventral onlay or 2-stage repair. Stricture recurrence occurred in 2 patients (15%), and postoperative complications of urtherocutaneous fistula, compartment syndrome, and glans dehiscence were seen in one patient each (7%) (44).

In 2016, a retrospective review analyzed four urethral reconstructive surgeries that used a transanal rectal mucosal graft (RMG) harvest to treat long segment, complex urethral strictures (40). All strictures were bulbopendulous, with a median length of 13.5 cm (10-21). One patient has stricture recurrence at ten months. No colorectal complications were seen (40).

The transanal minimally invasive surgery (TAMIS) approach was first described in 2010 to treat rectal pathology using a single-port transanal platform with laparoscopic instruments. The technique has been used to resection benign and malignant lesions with great success and minimal morbidity (45–50). TAMIS has also been used for a total mesorectal excision (TME) in cancer (47, 51–56). A successful robotic transanal minimally invasive surgery (R-TAMIS) approach was later described in 2012 to excise benign and malignant rectal masses and perform a transanal TME. Multiple successful series have been recorded since (45–49).

In 2019, Howard and colleagues described the R-TAMIS technique for urethral reconstruction (38). A posterior harvest is preferred due to its lower risk of peritoneal entry. Longer harvests of mucosa (up to 15 cm) are typically needed, so the widest site uninterrupted by valves is preferably selected. When harvesting RMG, considerations should be made for patients with prior anorectal surgeries and transgender patients who have previously undergone phalloplasty or vaginoplasty (38). The rectal harvest starts approximately 2 cm proximal to the dentate line in a distal to proximal fashion. The mucosa is measured, the tissue is scored with cautery, and the graft is raised using hydrodissection and subsequently excised and removed through the laparoscopic port (38).

In 2019, Howard and colleagues (38) showed in a series of six patients undergoing robotic rectal mucosal harvest that the R-TAMIS technique was a safe and feasible option for both cisgender and transgender patients requiring sizable amounts of graft tissue for lower tract stricture repair. No postoperative complications were seen, including bleeding, perforation, abscess, or obstruction, and all grafts were sufficient in size for repair. Graft length ranged from 7.5-15.0 cm (mean 11.4 cm), with a 3 cm width consistent throughout the series. Patients who previously underwent BMG harvesting subjectively self-reported less postoperative pain and a greater quality of life. All patients tolerated a regular diet within 12-24 hours after surgery and regained normal bowel function. Low numbers of long-term postoperative or bowel-related complications were reported at a median follow-up of 17 months; two patients (33%) developed recurrent stricture or stenosis (38).

### Colonic mucosal graft

Colonic mucosal grafts have also been described for use in urethral reconstruction (41, 42, 57). In this technique, 10-15 cm of the sigmoid colon is isolated from the intestinal tract along with a mesenteric pedicle, and continuity is restored with an end-to-end anastomosis (41). A full-thickness mucosal graft is harvested from the isolated portion of the bowel, which is then resected. Any excess submucosal tissue, fat, or muscle is removed from the graft to optimize subsequent vascularization. Next, a neourethra is created using an unstretched 10-17 x 2.5-3 cm segment of the colonic mucosa, tubularized over a 22Fr catheter using interrupted sutures. End-to-end anastomosis is then performed between the native urethra and neourethra (41).

In 2004, Xu and colleagues (41) followed 16 patients who received treatment for a long, complex urethra stricture with colonic mucosa. Urethral reconstruction was performed using 10-17 cm (median 13 cm) colonic mucosal grafts. One patient experienced a complication of meatal stenosis three months postoperatively and required reoperation. Otherwise, no significant complications were seen among the remaining patients at six months postoperative follow-up.

Long-term outcomes of colonic mucosal grafts used to treat long segment and complex ureteral strictures were studied in a retrospective review of 36 patients (42). The mean colonic mucosa graft was 15.1 cm (11-21 cm). Surgeries were completed without recurrence of stricture in 30 of 35 patients (85.7%); complications of meatal stenosis, bulbar or bulboembranous urethral stenosis, and anastomotic site strictures were seen in a total of 5 patients (13.3%) at a mean follow up of 53.6 months (26-94) (42). Colonic mucosal graft urethroplasty is a safe and feasible option for patients unable to undergo a BMG.

## Conclusion

The use of bowel plays a prominent role in treating urinary stricture disease. For upper tract stricture disease, appendiceal techniques are an effective and safe first-line treatment option. In using appendiceal grafts, there are minimal electrolyte disturbances and few side effects from the grafted tissue. The added advantage of its maintained blood supply and ability to create tension-free anastomoses makes it more favorable over other techniques. While the ileal ureter is still a valuable option, it is typically reserved for cases where harvesting the appendix is not viable or appendiceal length is insufficient for the magnitude of stricture.

For lower tract stricture repair, the RMG is an excellent, minimally invasive technique used in place of a BMG for longer urethral repairs for cisgender and transgender patients. The colonic mucosal graft technique is a valuable third-line treatment option for patients with long, complex urethral strictures. Though small retrospective series support the efficacy of this technique, robust prospective data is limited, and further research is needed.

## Author contributions

Conceptualization: HP, SE. Data Curation: SK, AK, HP. Investigation: SK, AK. Writing – original draft: SK. Writing – review & editing: SK, AK, HP, SE. All authors contributed to the article and approved the submitted version.
